# First Clinical Experiences with the Ultra-Fast Time-of-Flight BIOGRAPH One Next-Generation Hybrid PET/MRI System

**DOI:** 10.3390/diagnostics16030398

**Published:** 2026-01-27

**Authors:** Otto M. Henriksen, Kirsten Korsholm, Annika Loft, Johanna M. Hall, Annika R. Langkilde, Vibeke A. Larsen, Thomas S. Kristensen, Caroline Ewertsen, Frederikke E. Høi-Hansen, Patrick M. Lehmann, Karen Kettless, Flemming L. Andersen, Thomas L. Andersen, Ian Law

**Affiliations:** 1 Department of Clinical Physiology and Nuclear Medicine, Copenhagen University Hospital Rigshospitalet, 2100 Copenhagen, Denmarkian.law@regionh.dk (I.L.); 2 Department of Radiology, Copenhagen University Hospital Rigshospitalet, 2100 Copenhagen, Denmark; 3 Siemens Healthcare A/S, 2750 Ballerup, Denmark; 4 Research & Clinical Translation, Magnetic Resonance, Siemens Healthineers AG, 91052 Erlangen, Germany; 5 Department of Clinical Medicine, University of Copenhagen, 2200 Copenhagen, Denmark

**Keywords:** PET/MRI, hybrid imaging, PET/CT

## Abstract

**Objective:** We present the first clinical experience with the BIOGRAPH One next-generation PET/MRI system scanner, evaluating its performance for body and brain imaging in patients across multiple tracers. **Methods:** A total of 59 patients were scanned on the BIOGRAPH One PET/MRI following standard clinical PET/CT (n = 52) or first-generation PET/MRI (Biograph mMR, n = 7). Scans comprised 30 total body (TB), whole body (WB), or regional scans with [^18^F]FDG, and 29 brain scans with either [^18^F]FDG (n = 5), [^18^F]FE-PE2I (n = 10), [^18^F]FET (n = 4), or [^68^Ga]Ga-DOTATOC (n = 10). The PET image quality was visually assessed using a 5-point Likert scale (1 = very good to 5 = very bad) and compared with clinical scans acquired on either a current-generation digital PET/CT or a first-generation PET/MRI system, including evaluation of diagnostic concordance. PET quantification and image noise was compared in brain and WB/TB [^18^F]FDG PET scans. **Results:** PET image quality was rated as good or very good in 93% of scans with a median [inter-quartile range] score of 1.5 [1.5;2]. In 99% of cases, image quality was judged equal to or better than the clinical reference scan (median score 3 [2.5;3]). Diagnostic concordance was observed in 99% of readings. Imaging metrics revealed the anticipated regional bias in brain imaging, while no significant bias was observed in body imaging. Image noise was comparable to that observed with digital PET/CT and demonstrated superiority over first-generation PET/MRI despite potential degradation related to isotope decay in BIOGRAPH One PET/MRI acquisitions scans performed at the end of the imaging workflow. **Conclusions:** Within the study limitations related to sequential imaging, the BIOGRAPH One PET/MRI scanner demonstrated improved PET sensitivity and workflow potential over its first-generation predecessor, which may allow for broader clinical and research applications.

## 1. Introduction

Since the introduction of the first commercial integrated hybrid PET/MRI system in 2010 [1,2], a growing number of installations and numerous publications have demonstrated the value of simultaneous PET and MRI. Recent systematic reviews and meta-analyses have concluded that PET/MRI provides similar or superior diagnostic accuracy to PET/CT for a range of malignancies [3,4] including gynecological [5], head–neck [6], prostate [7], breast [8], lung [9], and multiple myeloma [10]. Further, international society practice guidelines have recently been published [11]. PET/MRI has also gained general acceptance for brain imaging as a convenient alternative to separate imaging sessions and is included in current practice guidelines for dementia [12], epilepsy [13], and brain tumors [14].

However, it has become increasingly evident that first-generation hybrid PET/MRI systems are falling behind in terms of MRI performance, PET detector technology, and software capabilities when compared to state-of-the-art stand-alone systems. The first commercial hybrid PET/MRI system (the Siemens Healthineers Biograph mMR, Forchheim, Germany) was equipped with analogue PET detectors lacking time-of-flight (TOF) capability and the MRI system was based on the Magnetom Verio 3T scanner introduced in 2007. Additionally, this system does not use the same software or sequence platform as the vendor’s existing MRI systems. In a PET/MRI user survey, responders raised concerns about the benefit of body imaging using PET/MRI compared to PET/CT, long acquisition times, attenuation correction challenges, and higher operating and maintenance costs as hurdles for daily use [15].

In 2024, Rigshospitalet in Copenhagen was the first clinical site worldwide to install the next-generation BIOGRAPH One PET/MRI system (Siemens Healthineers, Forchheim, Germany). The BIOGRAPH One integrates the current-generation Biograph Vision 600 PET/CT platform (introduced in 2019; Siemens Healthineers, Hoffman Estates, IL, USA) with the 3T MAGNETOM Vida MRI scanners (introduced in 2020; Siemens Healthineers, Forchheim, Germany). Notably, BIOGRAPH One provides an extended PET axial field of view (FOV) of 35.0 cm, compared with 26.3 cm for the Biograph Vision 600 PET/CT and 25.3 cm for the Biograph mMR PET/MRI, thereby enabling higher sensitivity and improved anatomical coverage. The integration of fast TOF resolution and reduced crystal cross-sectional dimensions enables shorter acquisition protocols, improves signal-to-noise ratio (SNR), and enhances image resolution. Clinically, these advancements allow for faster imaging workflows, improved lesion detectability, and potentially reduced patient discomfort during scanning

Following initial testing and software improvements, we conducted a clinical trial for conformity assessment from March to June 2025. Patients were scanned on the BIOGRAPH One PET/MRI following a standard-of-care clinical scan. These data enable a direct comparison of BIOGRAPH One PET/MRI performance with state-of-the-art digital PET/CT and first-generation PET/MRI systems.

In this study, we present our initial clinical experience with brain, whole body (WB), and regional PET imaging, illustrating examples across a range of tracers for diverse clinical indications. Additionally, we discuss potential applications of the enhanced capabilities of BIOGRAPH One PET/MRI in both clinical practice and research settings.

## 2. Materialsand Methods

The aim of the study was to include a total of 60 adult patients referred to for WB (n = 30) and brain PET (n = 30). The study was approved by both The Medical Research Ethics Committees and The Danish Medicines Agency under Medical Device Regulation article 62 (Case nr: 2401738) and performed according to the Helsinki Declaration. All patients gave informed oral and written consent prior to the PET/MRI scan on the BIOGRAPH One PET/MRI.

All scans on the BIOGRAPH One PET/MRI were performed directly after a clinical scan on one of our standard FOV PET/CT (Biograph Vision 600), long-axis FOV (LAFOV, Biograph Vision Quadra) PET/CT (both Siemens Healthineers, Hoffman Estates, IL, USA), or first-generation PET/MRI (Biograph mMR) systems. Study scans were performed as WB (vertex to mid-femur), total body (TB, vertex to toe), or regional [^18^F]FDG PET scans or brain scans with tracers and indications as summarized in Table 1. PET acquisition and reconstruction protocols are summarized in Table 2. Long-lived tracers with relatively stable tracer binding were selected for the study to visualize pathology on delayed imaging.

PET acquisitions continued throughout the duration of all MRI sequences, thus exceeding the pre-defined minimum target duration for single-bed acquisition (Table 1). For multi-bed acquisitions, an overlap of 6.3 cm was applied resulting in an effective axial coverage of 31.9 cm and allowing WB and TB imaging to be performed by 4 and 7 (or 8) bed positions, respectively. PET imaging was reconstructed using vendor-provided MRI segmentation-based attenuation correction (MRAC), including a bone model. PET reconstruction parameters were chosen to match those of our clinical standard FOV PET/CT protocols

The MRI protocols were designed to simulate fast clinical protocols with both pre- and post-contrast imaging for both brain and female pelvis. All neuro and head and neck scans were acquired with a 20-channel head and neck coil (Biomatrix Head/Neck Pro PET/MR). Either one or two flexible 24-channel body coils (Biomatrix Contour XL) and a 32-channel spine coil (Biomatrix Spine Pro PET/MR) were used for non-brain imaging. Due to study restrictions, no MRI contrast was administered. Details of MRI protocols are provided in Supplementary Table S1.

### 2.1. Image Reading

Imaging was rated for PET and MRI image quality by a total of eight readers: four experienced nuclear medicine physicians (two readers evaluating brain imaging, and two readers evaluating TB/WB/regional [^18^F]FDG PET) and four experienced radiologists evaluating MRI (two neuro-radiologists and two whole-body radiologists evaluating brain and body imaging, respectively). As the shorter MRI protocols and sequences in the study were not aligned with local preferences, MRI quality scores will be biased negatively. Therefore, the present analysis focuses on the results of the PET readings.

First, images were evaluated using a 5-point Likert scale for overall image quality (1 = very good to 5 = very bad), image noise (1 = none to 5 = very strong), contrast (1 = very good to 5 = very bad), and resolution (1 = very good to 5 = very bad) with a score of 3 indicating acceptable quality. Subsequently, PET image quality was compared to that of the clinical PET/CT or PET/MRI (1 = very good to 5 = very bad) with a score of 3 indicating equal quality, and the diagnostic comparability of the two scans was assessed. Readers were not blinded to the scanner system and no inter-reader agreement analysis was performed.

### 2.2. Image Analysis

Selected metrics were obtained from [^18^F]FDG PET images to compare the image noise and PET quantification of BIOGRAPH One with that of the clinical PET/CT or PET/MRI scan. For the brain, the standardized uptake value (SUV) in the frontal cortex, occipital cortex, and basal ganglia normalized to the cerebellum (SUVr) was determined using the syngo.via DB Comparison tool (Scenium, version VE70A, Siemens Healthineers, Forchheim, Germany). In non-brain scans, the peak SUV (highest average 1 mL sphere) was determined in 10 patients with [^18^F]FDG avid tumors. In each patient, the lesion or component of the lesion with the highest SUV was included for analysis. Image noise was assessed using the coefficient of variation (COV) in a 5–10 mL volume of interest (VOI) in the liver of WB/TB [^18^F]FDG scans and in a 2 mL VOI in the white matter of brain [^18^F]FDG scans.

### 2.3. Statistics

Values are reported as the median [inter-quartile range, IQR]. Liver COV and lesion SUVpeak values are compared by Wilcoxon signed rank test. For brain metrics, the number of observations was considered too low for formal statistical testing.

## 3. Results

Patient recruitment and scans were completed successfully for all categories except for [^18^F]FET, where only 4 of 5 planned patients were recruited within the study period. The median time from the start of the clinical scan to the start of the BIOGRAPH One PET/MRI study scan was 33 min [IQR: 26;36 min]. Median delay, duration, and average quality scores of individual scan protocols are provided in Table 3.

### 3.1. Image Reading

Based on 59 averaged paired readings (118 in total), the median score was 1.5 [IQR: 1.5;2] for image quality with 93% rated as good or very good. The median scores were 2 [IQR: 2;2] for image noise, 1.5 [IQR: 1.5;2] for contrast, and 1.5 [IQR: 15;2] for resolution. When comparing image quality to the clinical scan, the median (range) score was 3 [IQR: 2.5;3] with 99% rated as equal or better. The average quality ratings of MRI for individual imaging protocols are provided in Supplementary Table S2. In 117 of 118 (99%) of readings, diagnostic comparability was obtained. One single scan rated as non-comparable was related to apparent tracer uptake in bone in a brain FDG scan due to error in the segmentation-based MRAC.

**Figure 1:**
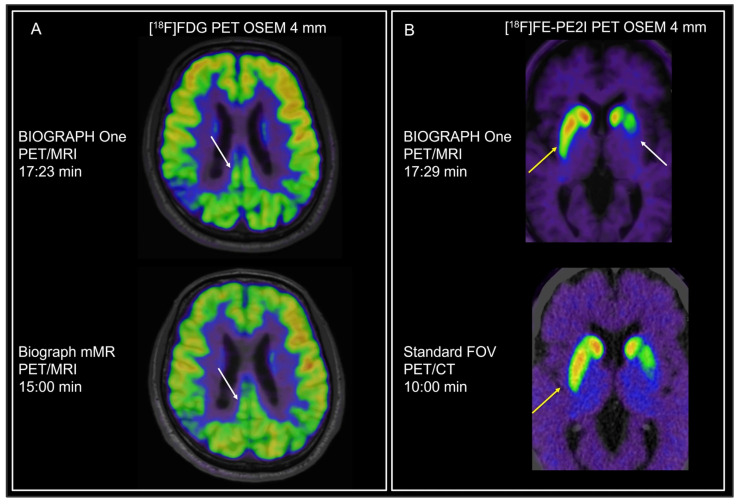
Neurodegenerative disease. (**A**) [^18^F]FDG PET imaging in a patient where Alzheimer’s disease was suspected. Imaging from both PET/MRI systems reveal marked parietal hypometabolism also involving the mesial parietal cortex and the cingulate gyrus typical of AD, more pronounced in the right hemisphere. Note the better delineation of the cortical structures on BIOGRAPH One PET/MRI (white arrows) at a similar duration of PET acquisition compared to Biograph mMR PET/MRI. (**B**) [^18^F]FE-PE2I imaging in a patient where Parkinson’s disease was suspected. Images show reduced dopamine transporter binding, predominantly in the posterior putamen and more pronounced on the left side corresponding to right-sided symptoms. MRI reveals no underlying structural pathology and supports primary nigrostriatal degeneration. The morphology could not be assessed on low-dose CT and warranted further imaging. Note also thinning of the posterior putamen on the right side not visually evident on PET/CT (yellow arrows), possibly because of higher resolution allowing better anatomical delineation or earlier identification of nigrostriatal dysfunction, or alternatively reflecting redistribution on late imaging.

**Figure 2:**
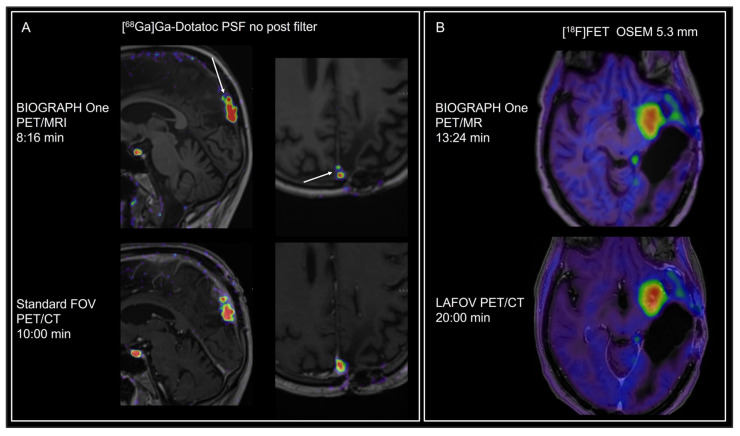
Short-duration imaging of brain tumors. (**A**) [^68^Ga]Ga-DOTATOC imaging in a patient with residual meningioma WHO grade 2 in the sagittal sinus. Images are scaled to the same SUV range. Tumor SUV values and tumor volume (SUV > 2.5) deviated by <5%. Note the better spatial separation of the smaller superior tumor component (white arrows) on BIOGRAPH One PET/MRI compared to standard FOV PET/CT despite the shorter acquisition time, although later imaging may also have improved the signal-to-noise ratio. (**B**) [^18^F]FET imaging in glioma. Patient with multifocal progressive isocitrate dehydrogenase–mutant astrocytoma (WHO grade 3). Total tumor-to-background ratios (TBR) and metabolically active tumor volume (TBR > 1.6) differed by less than 3%. Comparable image quality was achieved with clear delineation of multiple tumor components despite a shorter acquisition time. Long axial field-of-view (LAFOV) PET/CT is shown fused with prior contrast-enhanced T1-weighted MRI.

**Figure 3:**
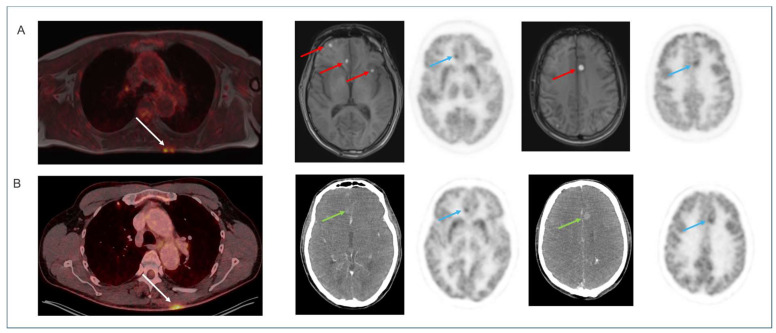
Total body imaging. Staging [^18^F]FDG PET in a patient with a surgically removed melanoma on the back. BIOGRAPH One PET/MRI (**A**) and PET/CT (**B**) from vertex to toe showed metabolic activity at the site of resection, interpreted as surgery related (white arrows), but also multiple metabolically active metastases in the brain (blue arrows) with contrast enhancement on CT (green arrows). Note that T1-weighted Dixon identified additional hyperintense foci (red arrows) reflecting melanin containing metastases not detected on CT or PET.

**Figure 4:**
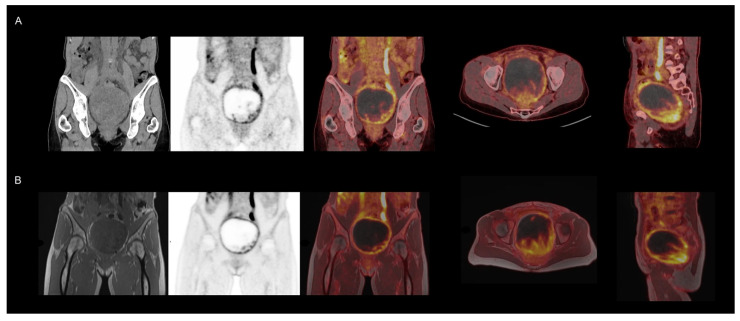
Regional focused imaging—female pelvis. Suspected recurrent disease in a patient with prior surgery for carcinosarcoma in the right ovary/adnexa. PET/CT (**A**) and BIOGRAPH One PET/MRI (**B**) revealed a heterogeneously metabolically active tumor suggesting recurrence, subsequently confirmed by surgery. PET acquisition was performed using continuous bed motion at 1.5 mm/s, corresponding to approximately 3 min per field of view (FOV) for PET/CT, compared to 21 min for a single-bed PET/MRI acquisition.

**Figure 5:**
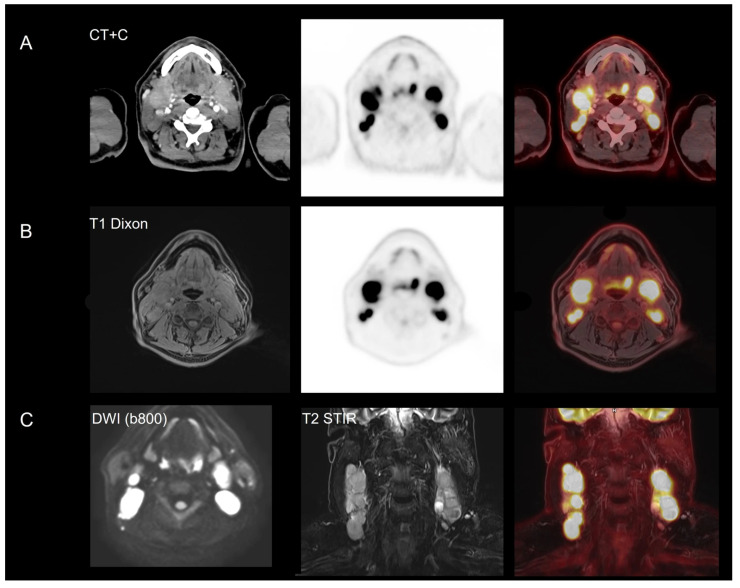
Regional focused imaging—head/neck. Patient with a swelling on both sides of the neck, but no clinical signs of a primary tumor. Both PET/CT (**A**) and BIOGRAPH One PET/MRI (**B**) revealed the primary tumor in the base of the tongue, in addition to metabolically active lymph nodes on both sides of the neck. Biopsy confirmed a tongue base cancer, squamous cell carcinoma, T2N3M0, HPV+. In the lower row (**C**), DWI shows concordant diffusion restriction corresponding to both [^18^F]FDG avid lymph nodes and the primary tumor. Coronal T2 STIR and [^18^F]FDG show enlarged metastatic cervical lymph nodes. PET acquisition was performed using continuous bed motion at 1.5 mm/s, corresponding to approximately 3 min per field of view (FOV) for PET/CT, compared to 20 min for a single-bed PET/MRI acquisition.

Examples of the different tracers and applications are shown in Figure 1, Figure 2, Figure 3, Figure 4 and Figure 5. MRI identified additional findings in two patients. In one case, an occluded internal carotid artery—undetectable on low-dose CT—was revealed on MRI. In another case, MRI detected additional cerebral metastases compared to contrast-enhanced CT and PET in a patient with malignant melanoma (Figure 3). Conversely, a 7 mm non-FDG-avid pulmonary nodule was reported on CT in one patient, but was not visible on MRI. No other clinically relevant discrepancies were noted.

### 3.2. Image Metrics

The body and brain PET metrics are summarized in Table 4. Compared to PET/CT and first-generation PET/MRI, the combined median [^18^F]FDG ROI SUVr values from BIOGRAPH One PET/MRI tended to be higher in the frontal cortex (+13.7%), in the occipital cortex (+4.6%), and in the basal ganglia (+8.1%), resulting in noticeable regional differences in the scaling of 3/5 statistical surface projection maps.

**Figure 6:**
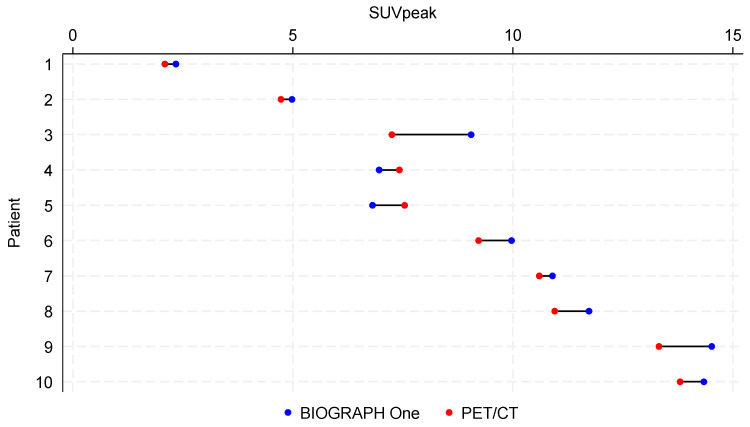
Dumbbell plot of SUVpeak from metabolic active tumors from 10 different patients from BIOGRAPH One PET/MRI and PET/CT. Patients are ordered according to increasing SUVpeak from PET/CT. PET/CT was performed on standard FOV (n = 5) or LAFOV (n = 5) systems.

The SUVpeak values from WB/TB and regional scans were higher, with a median difference of 6.2% in images from BIOGRAPH One PET/MRI compared to PET/CT (Figure 6), although the difference did not reach statistical significance (*p* = 0.08).

**Figure 7:**
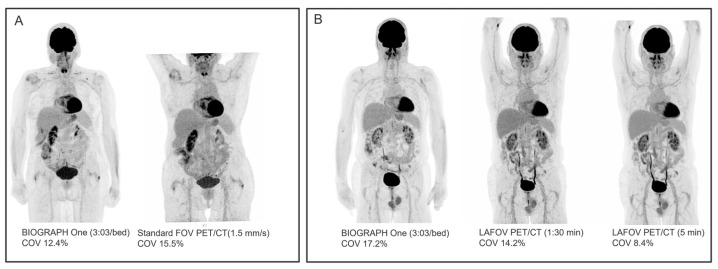
WB image quality. (**A**) WB [^18^F]FDG PET scan of patient with follicular lymphoma showing complete metabolic remission following 6 cycles of chemotherapy. Scan on BIOGRAPH One PET/MRI was performed 87 min p.i. (duration 20:28). COV is comparable between BIOGRAPH One PET/MRI and standard FOV PET/CT. (**B**) TB [^18^F]FDG PET following surgical removal of malignant melanoma (only vertex to femur shown). Scan on BIOGRAPH One PET/MRI was performed 87 min p.i. (duration 33:01).

The image noise (Figure 7 and Table 4) assessed as COV in the liver of WB and TB scans showed a median COV of 17.2% from BIOGRAPH One PET/MRI (n = 15) compared to 15.5% from standard FOV PET/CT (n = 7, *p* = 0.128 for difference) and 8.1% from the LAFOV PET/CT (5 min) (n = 8, *p* = 0.012 for difference). For the brain FDG [^18^F]FDG scans, the median COV in WM was 9.6% (n = 5) compared to 11.7% from a 15 min first-generation PET/MRI scan (n = 4), and also lower (8.5% vs. 10.7%) compared to a 10 min clinical scan on standard FOV PET/CT (n = 1).

## 4. Discussion

In this report, we present our initial clinical experiences with imaging across a range of tracers and pathologies using the world’s first clinical installation of the next-generation BIOGRAPH One PET/MRI system.

Compared directly with its predecessor, the BIOGRAPH One offers markedly improved performance. In addition to longer axial FOV, preliminary PET characterization (data presented at ISMRM annual meeting, 2025, Honolulu, HI, USA, article in preparation) shows a >50% percent increase in sensitivity (14.1 cps/kBq vs. 22.7 cps/kBq), approximately 1 mm improvement in image resolution (4.3 mm vs. 3.4 mm), and a TOF time resolution below 190 ps. In contrast, the Biograph mMR PET/MRI does not have TOF capability. The MRI component is based on the MAGNETOM Vida platform with compatible software and a gradient amplitude increase from 45 mT/m to 60 mT/m.

### 4.1. PET Image Quality

The scanner demonstrates enhanced PET imaging performance compared to the first-generation Biograph mMR system, offering image quality similar to that of leading digital PET/CT systems. Only a few added or missed findings were observed. As a reminder, our study was not designed to assess potential diagnostic gains or superiority.

The COV measurements were similar or lower than our reference systems with the exception of the LAFOV PET/CT (5 min scan). However, COV values are a limited measure of scanner performance, due to decreased tissue activity from physical and biological decay in delayed images and differences in scan duration and reconstruction parameters.

The improved sensitivity may permit shorter scans or reduced administered activity compared to Biograph mMR PET/MRI. Although not equivalent to standard longer MRI protocols, shorter protocols may be attractive in patients who are not able to complete a standard clinical protocol. Combining accelerated MRI with MRI-guided PET reconstruction [16] could permit ultra-fast low-dose imaging, e.g., a 5 min brain protocol at less than half standard activity. Alternatively, acquiring PET data during the full duration of a standard MRI protocol could allow for a significant dose reduction. Low-dose imaging is of particular importance in children, pregnant women, patients with cancer-prone syndromes, and healthy volunteers.

### 4.2. PET Quantification

The systematically higher SUVr values in brain [^18^F]FDG are consistent with the well-known bias in segmentation-based MRAC which may adversely impact the image reading. This is a particular issue using statistical surface projections comparing patient data to normal controls from PET/CT, or when using a reference region for calculation of uptake metrics. At our institution, we have relied on an in-house developed deep learning Dixon-based synthetic CT for PET/MRI MRAC showing superior PET quantification accuracy [17,18].

**Figure 8:**
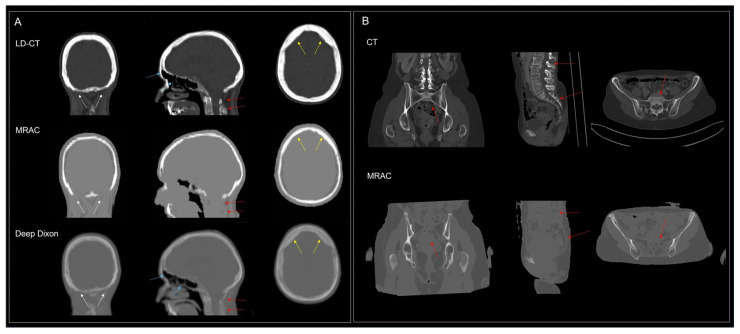
Comparison of atlas-based MRAC µ-maps with low-dose CT of the brain (**A**) and female pelvis (**B**). Compared to CT, segmentation-based MRAC failed to detect air filled cavities in the frontal sinus and nasal cavity (blue arrows), bone in the spine and the sacrum (red arrows), bone in the posterior fossa (white arrows), and hyperostosis in the frontal bone (yellow arrows). Data from the patient in panel (**A**) was obtained in clinical routine after CE labeling, and the results of in-house deep learning Dixon-based synthetic CT (work in progress) are shown for comparison.

The SUVpeak values in FDG-avid lesions in body imaging were higher, although not statistically significant, and likely related to later imaging times. Some regional bias may be derived from the segmentation-based MRAC (Figure 8).

By visual analysis, tracer distribution differed insignificantly for [^18^F]FET (Figure 2) and [^68^Ga]Ga-DOTATOC was constant with mean SUV differing by <5%.

### 4.3. Brain Applications

At our institution PET/MRI is the preferred one-stop-shop imaging modality for memory clinic patients with >800 exams performed yearly. The BIOGRAPH One PET/MRI will allow for shorter protocols or advanced imaging sequences within a clinically acceptable scan duration. Other key applications are the use of amino acid PET imaging of gliomas [19] and [^68^Ga]Ga-DOTATOC, although attention to effects of the MRAC method is of key importance [18].

The longer axial FOV of 35 cm permits better coverage of large arteries and will provide an image-derived input function less affected by partial volume effects [20] for quantitative kinetic modeling in research and the clinic, e.g. for cerebrovascular flow reserve measurements with [^15^O]H_2_O PET [21].

### 4.4. Non-Brain [^18^F]FDG Oncology Applications

For non-brain imaging, [^18^F]FDG PET/CT represents a robust and versatile modality with wide applications in oncology, infection/inflammation, and cardiac imaging. Hybrid PET/MRI may be employed as either focused regional imaging when both PET and diagnostic MRI are indicated, as a low-radiation alternative to WB PET/CT, or as a combination of focused regional imaging for tumor staging (T) and locoregional lymph nodes (N) and WB screening for distant metastatic disease (M).

The longer axial FOV allows faster multi-bed imaging. For example, a WB (vertex to mid femur) PET/MRI scan can be accomplished within 20 min or less with all-purpose MRI sequences such as Dixon T1, T2, and DWI (diffusion-weighted imaging). Such a minimal protocol may be comparable to PET with low-dose CT and applicable for screening or WB staging of diseases such as lymphoma [11]. A recurring concern for the use of PET/MRI for WB imaging is the lower sensitivity for detection of non-FDG-avid small lung nodules <1 cm with a detection rate reported as low as 12%, although with no impact on patient management in a per patient analysis [22]. The detection rate may be improved by adding Ultrashort Echo (UTE) or Zero Echo Time (ZTE) sequences and using respiratory gating, but at the expense of longer scanning. In the present study a single non-avid pulmonary nodule was not identified on MRI. A low-dose CT of the thorax three months later displayed a stationary benign-looking flat-shaped 7 mm calcification in the left lung.

Significant clinical value can be obtained using similar basic protocols that minimize radiation exposure or realize the full diagnostic potential of MRI when combined with focused regional MRI, e.g., in pelvic diseases or head–neck cancer as in the present study. Rather than simply combining standard MRI protocols with PET, MRI sequences should be designed to provide complementary diagnostic information, e.g., by improving anatomical information or by disqualifying benign [^18^F]FDG-avid lesions, while avoiding redundancy. The limitations of segmentation-based MRAC for the identification of bone (Figure 6) remain a concern for PET for the assessment of lesions adjacent to bone.

### 4.5. MRI

The clinical performance of the integrated MRI system is expected to be comparable to that of the current 3T MAGNETOM Vida. Although only fast protocols—shorter than our standard protocols—were utilized in this study, MRI still provided additional diagnostic value by revealing an occluded internal carotid artery in one patient and detecting additional brain metastases in another. The system was equipped with a 20-channel head/neck coil, and further improvements in image quality are anticipated with the planned implementation of a 32-channel coil.

The enhanced MRI capabilities may be particularly valuable in applications such as in epilepsy surgery planning, where high-quality MRI and PET are essential for the detection of subtle abnormalities such as heterotopias and cortical dysplasias.

### 4.6. Limitations

This study has several limitations and does not constitute a formal validation of the system’s clinical performance. Rather, it serves as an early indication of its potential and highlights issues to be addressed following CE approval for clinical use. Sequential imaging may have introduced bias in perceived image quality due to improved signal-to-background contrast in delayed acquisitions, despite lower count density resulting from isotope decay. Additionally, selection bias may have occurred as the study population included relatively healthier patients. Finally, comparisons were limited to systems from the same vendor, and no evaluation against PET/CT or PET/MRI platforms from other manufacturers was performed.

## 5. Conclusions

The BIOGRAPH One PET/MRI system demonstrates strong potential for delivering state-of-the-art PET imaging quality comparable to current PET/CT systems, while expanding the range of possible clinical indications. Its advanced design may enable reductions in either scan time or administered activity relative to its predecessor, improving efficiency and patient safety. MRI-based attenuation correction remains a critical area of focus of attention for accurate PET quantification.

## Figures and Tables

**Table 1:** Overview of study scans, tracers, and indications.

Protocol	Tracer	Patients, n	Indication
Multi -bed			
Whole body, single pass ^a^	[^18^F]FDG	5	Lymphoma or sarcoma
Whole body, multi pass ^b^	[^18^F]FDG	5	Lymphoma or sarcoma
Total body (vertex to toe)	[^18^F]FDG	5	Melanoma, sarcoidosis, or sarcoma
Single-bed			
Head/neck	[^18^F]FDG	5	Head and neck squamous-cell carcinoma
Female pelvis	[^18^F]FDG	10	Cervical and ovarian cancer
Brain neuro	[^18^F]FDG	5	Dementia
Brain neuro	[^18^F]FE-PE2I	10	Parkinson’s disease/Parkinsonism
Brain onco	[^18^F]FET	4	Glioma or pituitary adenoma
Brain onco	[^68^Ga]Ga-DOTATOC	10	Meningioma

^a^ scans performed position-wise; ^b^ scans performed sequence-wise. Abbreviations: FDG = fluorodeoxyglucose, FE-PE2I = (E)-N-(3-iodoprop-2-enyl)-2β-carbofluoroethoxy-3β-(4′-methyl-phenyl) nortropane, FET = fluoroethyl-L-tyrosine, Ga-DOTATOC = DOTA-DPhe1-Tyr3-Octreotide.

**Table 2:** PET acquisition and reconstruction.

Clinical Scan Protocol	Activity	Standard FOV PET/CT	LAFOV PET/CT	mMR PET/MRI	BIOGRAPH One PET/MRI
	Scan Start				
Whole body [^18^F]FDG	3 MBq/kg 60 min p.i.	1.5 mm/s ^a^ PSF + TOF 2 mm Gauss 4 iteration/5 subsets	5 min PSF + TOF 2 mm Gauss 4 iteration/5 subsets	-	WB/TB: 3:00/3:03 min ^b^ Head/neck: 4:00/20:07 Female pelvis: 4:00/23:02 PSF + TOF 2 mm Gauss 3 iteration/5 subsets
Brain neuro [^18^F]FDG	200 MBq 40 min p.i.	OSEM + TOF 4 mm Gauss 12 iterations/5 subsets	-	15 min OSEM 4 mm Gauss 4 iterations /21 subsets	10:00/17:26 min ^b^ OSEM + TOF 4 mm Gauss 12 iterations/5 subsets
Brain neuro [^18^F]FE-PE2I	200 MBq 30 min p.i.	10 min OSEM + TOF 4 mm Gauss 12 iterations/5 subsets	-	10 min PSF 4 mm Gauss 4 iterations/21 subsets	10:00/18:07 min ^b^ OSEM + TOF 4 mm Gauss 12 iterations/5 subsets
Brain onco [^18^F]FET	200 MBq 20 min p.i.	20 min OSEM + TOF 2 mm Gauss 8 iterations/5 subsets	20 min OSEM + TOF 5.3 mm Gauss 4 iterations/5 subsets	20 min OSEM 5 mm Gauss 4 iterations/21 subsets	7:00/8:19 min ^b^ OSEM + TOF 5 mm Gauss 3 iterations/5 subsets
Brain onco [^68^Ga]Ga-DOTATOC	100 MBq 50 min p.i.	10 min PSF + TOF No post filter 12 iterations/5 subsets	-	-	7:00/8:14 min ^b^ PSF + TOF No post filter 8 iterations/5 subsets

^a^ Continuous table motion (2.2 mm/s for legs/arms); ^b^ predefined minimum target/actual duration.

**Table 3:** Main results.

Protocol	Delay (min) ^a^	Median Exam Duration (min) ^b^	Median [IQR] PET Quality Score ^c^	Median [IQR] MRI Quality Score ^c^	Median [IQR] Diagnostic Score ^d^
Multi-bed ([^18^F]FDG)					
Whole body, single pass ^c^	41:30	20:28	1.3 [1.1;1.3]	2.9 [2.8;3]	2.5 [2;3]
Whole body, multi-pass ^d^	33:08	27:29	1.6 [1,4;1.8]	2.9 [2.9;3]	2.5 [2;2.5]
Total body (vertex to toe)	28:40	35:32	1.6 [1.6;2.0]	2.8 [2.8;3]	2.5 [2;2.5]
Single-bed					
Head/neck ([^18^F]FDG)	27:40	28:22	1.9 [1.8;2.0]	2.8 [2.7;2.9]	2 [2;2]
Female pelvis ([^18^F]FDG)	29:17	28:38	1.6 [1.5;1.9]	2.9 [2.5;2.9]	2.5 [2;3]
Brain neuro ([^18^F]FDG)	34.20	22:49	1.6 [1.6;1.6]	2.9 [2.7;2.9]	3 [2.5;3]
Brain neuro ([^18^F]PE2I)	40:53	24:02	1.7 [1.6;1.8]	2.6 [2.6;2.8]	3 [3;3]
Brain onco ([^18^F]FET)	41:55	12:52	1.6 [1.6;1.7]	2.2 [2.1;2.2]	3 [3;3]
Brain onco ([^68^Ga]Ga-DOTATOC)	28:41	11:47	2.0 [1.9;2.3]	2.7 [2.6;2.8]	3 [3;3]

^a^ Time from start of clinical scan to start of study scan; ^b^ from start to stop of scan; ^c^ average of scores from two readers of image quality, noise, contrast, and resolution; ^d^ for PET vs. clinical scan.

**Table 4:** [^18^F]FDG body and brain PET metrics.

	BIOGRAPH One	Biograph mMR	Standard FOV PET/CT	LAFOV PET/CT
Brain				
COV WM (%)	9.6 [9;11.5]	11.7 [10.9;16.0]	10.7	-
SUVr Frontal	1.22 [1.19;1.38]	1.06 [0.96;1.20]	1.21	-
SUVr Occipital	1.34 [1.18;1.45]	1.13 [1.08;1.41]	1.26	-
SUVr Striate	1.38 [1.24;1.49]	1.22 [1.10;1.36]	1.28	-
Body				
COV liver (%)	17.2 [16.5;17.3]	-	15.5 [13.2;16.2]	8.1 [7.3;8.8] ^‡^
SUVpeak [a.u.]	9.51 [6.81;11.73]	-	7.54 [7.42;9.22]	10.6 [7.25;10.95]

All values are median [IQR], COV = coefficient of variation, SUVr = standardized uptake value normalized to cerebellum, SUVpeak = peak SUV, WM = white matter. ^‡^ *p* < 0.01 vs. BIOGRAPH One. Note that *p*-values are not reported for brain metrics as the number of observations are too low.

## Data Availability

The datasets presented in this article are not readily available because data were acquired in a sponsored trial. Requests to access the datasets should be directed to the sponsor.

## Supplementary Materials

The following supporting information can be downloaded at https://www.mdpi.com/article/10.3390/diagnostics16030398/s1, Supplementary Table S1. MRI protocols; Supplementary Table S2. Rating scores by protocol and criterion..
