# Supplementary material for: First Clinical Experiences with the Ultra-Fast Time-of-Flight BIOGRAPH One Next-Generation Hybrid PET/MRI System
Source: Diagnostics (Basel). 2026 Jan 27;16(3):398. doi: 10.3390/diagnostics16030398 (PMC12896713; doi:10.3390/diagnostics16030398)
Supplement: Supplementary file 1 [file diagnostics-16-00398-s001.zip › diagnostics-4070691-supplementary.pdf]

Supplemental Data

First clinical experiences with the ultra-fast time-of-flight BIOGRAPH One next generation hybrid PET/MRI system.

Suppl. Methods

Inclusion criteria:

- Adult test subjects for whom a PET-MRI examination is indicated and will be performed, or
- Test subjects for whom a PET-CT examination is indicated and will be performed,
- Test subjects of legal age who have given their written informed consent to participate in the clinical investigation,
- Written consent of the patient to be informed in the event of suspected clinically relevant incidental findings,
- Written consent to the disclosure of a clinically relevant incidental finding to a subsequent physician (release from the duty of confidentiality),
- The patient's written consent that the recorded image data may be made available to Siemens Healthcare A/S in pseudonymized form,
- Test subjects and test subjects whose weight does not exceed the permissible weight limit of the patient table (200 kg).

Exclusion criteria:

- Metallic foreign bodies/implants and electronic devices that are not safe to scan at 3 Tesla MRI systems (e.g. pacemakers, heart valves, neurostimulators, metal splinters)
- Pregnancy: The exclusion of study participants of childbearing potential is carried out based on a positive pregnancy test.
- Persons who indicate an increased sensitivity to loud noises
- Persons who are incapable of giving consent

Suppl. Table S1. MRI protocols

**Protocol**	**Brain Neuro**		**Brain Onco**		**TB/WB**	**per bed**	**WB 4 bed**	**TB 7 bed**	**Head/neck**		**Female**	
**Sequences**	Localizer	00:14	Localizer	00:14	Localizer		00:26	00:43	Localizer	00:13	Localizer	00:13
	MRAC	00:53	MRAC	00:14	T1 MRAC	00:15	01:00	01:45	T1 MRAC	00:56	T1 MRAC	00:56
	T1 MPRAGE	03:59	T1 sag	01:00	T1 Huge [a]	01:02	02:04	02:04	T1 Huge	01:02	T1 Huge	01:02
	T2 TSE FLAIR	02:52	T2 FLAIR tra	01:36	T2 Haste Tra	00:15	01:00	01:45	T2 QTSE sag	01:20	T2 QTSE tra	01:09
	DWI	00:34	DWI	00:38	DWI	01:11	04:44	08:16	T1 QTSE cor	01:45	T2 QTSE Stir cor	01:07
	T2 SWI	02:50	T2 TSE tra	00:43					T2 QTSE Stir cor	01:27	T2 QTSE sag	02:07
	T2 TSE	01:39	T1 MPRAGE post	02:50					T2 blade tra	02:50	T2 QTSE sax	01:54
	T1 MPRAGE post	03:59							DWI	02:54	T1 Vibe Dixon tra [b]	01:12
									T2 QTSE cor	01:37	DWI	01:56
											T1 Vibe sax dyn [c]	03:04
											T1 Vibe Dixon tra	00:56
**Total**		17:00		07:15			09:14	14:33		14:04		15:36
**Total, without localizer**		16:46		07:01			08:48	13:50		13:51		15:23

[a] extended coverage transverse for arms/hips, [b] only for ovaries protocol, [c] dynamic imaging during contrast bolus

Suppl. Table S2. Rating scores by protocol and criterion

	Onco Brain [^{68}Ga]Dotatoc (N = 10)	Brain Onco [^{18}F]FET (N = 4)	Brain Neuro [^{18}F]FDG (N = 5)	Brain Neuro [^{18}F]PE2I (N = 10)	Total body [^{18}F]FDG (N = 5)	WB multi-pass [^{18}F]FDG (N = 5)	WB single-pass [^{18}F]FDG (N = 5)	Female pelvis [^{18}F]FDG (N = 10)	Head-neck [^{18}F]FDG (N = 5)
**PET scores**									
Image Quality	2.0 (1.5, 2.5)	1.5 (1.5, 1.8)	1.5 (1.5, 1.5)	1.5 (1.5, 1.5)	1.5 (1.5, 2.0)	1.5 (1.5, 2.0)	1.0 (1.0, 1.0)	1.5 (1.0, 1.5)	2.0 (1.5, 2.0)
Noise	2.5 (2.0, 2.5)	2.0 (2.0, 2.0)	2.0 (2.0, 2.0)	2.0 (2.0, 2.5)	2.0 (2.0, 2.0)	2.0 (2.0, 2.0)	2.0 (1.5, 2.0)	2.0 (2.0, 2.0)	2.0 (2.0, 2.0)
Contrast	2.0 (1.5, 2.0)	1.5 (1.5, 1.5)	1.5 (1.5, 1.5)	1.5 (1.5, 1.5)	1.5 (1.5, 2.0)	1.5 (1.0, 1.5)	1.0 (1.0, 1.0)	1.5 (1.5, 2.0)	2.0 (1.5, 2.0)
Resolution	2.0 (1.5, 2.0)	1.5 (1.5, 1.5)	1.5 (1.5, 1.5)	1.5 (1.5, 1.5)	1.5 (1.5, 2.0)	1.5 (1.0, 1.5)	1.0 (1.0, 1.0)	1.5 (1.5, 2.0)	2.0 (1.5, 2.0)
**MRI scores**									
Image Quality	2.7 (2.6, 2.8)	2.2 (2.2, 2.3)	3.0 (2.8, 3.1)	2.8 (2.6, 2.9)	2.8 (2.8, 3.0)	2.8 (2.8, 3.0)	3.0 (2.8, 3.0)	2.9 (2.5, 3.0)	2.9 (2.8, 3.0)
Noise	2.2 (2.1, 2.3)	1.8 (1.7, 1.8)	2.5 (2.4, 2.8)	2.2 (2.1, 2.4)	2.8 (2.8, 3.0)	3.0 (2.8, 3.0)	2.8 (2.8, 2.8)	2.8 (2.6, 2.9)	2.6 (2.5, 2.7)
Contrast	3.0 (2.9, 3.2)	2.4 (2.2, 2.6)	3.1 (3.0, 3.2)	3.0 (2.8, 3.2)	2.8 (2.8, 3.0)	2.8 (2.8, 2.8)	2.8 (2.7, 3.0)	2.8 (2.5, 2.9)	2.8 (2.7, 3.0)
Resolution	2.7 (2.6, 3.1)	2.2 (2.2, 2.2)	2.7 (2.7, 2.8)	2.7 (2.5, 2.8)	2.8 (2.8, 3.0)	3.0 (2.8, 3.0)	2.8 (2.8, 2.8)	2.8 (2.6, 2.9)	2.9 (2.8, 2.9)

All values are median (IQR) of average scores of two readers. Rating scale for image quality, contrast and resolution: 1= very good., 2 = good, 3 acceptable, 4 =bad, 5= very bad. Rating scale for noise: 1= none,. 2= weak, 3= medium, 4= strong, 5= very strong.
